# Superantigen profiles of *emm *and *emm*-like typeable and nontypeable pharyngeal streptococcal isolates of South India

**DOI:** 10.1186/1476-0711-11-3

**Published:** 2012-02-02

**Authors:** Thangarajan Durai Anand, Thangamani Rajesh, Jeyaprakash Rajendhran, Paramasamy Gunasekaran

**Affiliations:** 1 Department of Genetics, Centre for Excellence in Genomic Sciences, School of Biological Sciences, Madurai Kamaraj University, Madurai-625 021, India; 2 Department of Microbial Engineering, College of Engineering, Konkuk University, 1, Hwayang-dong, Gwangjin-gu, Seoul, 143 701. Korea

**Keywords:** Pharyngeal streptococci, *emm *typing, superantigen profiling

## Abstract

**Background:**

The major virulence factors determining the pathogenicity of streptococcal strains include M protein encoded by *emm *and *emm*-like (*emmL*) genes and superantigens. In this study, the distribution of *emm, emmL *and superantigen genes was analyzed among the streptococcal strains isolated from the patients of acute pharyngitis.

**Methods:**

The streptococcal strains were isolated from the throat swabs of 1040 patients of acute pharyngitis. The *emm *and *emmL *genes were PCR amplified from each strain and sequenced to determine the *emm *types. The dot-blot hybridization was performed to confirm the pathogens as true *emm *nontypeable strains. The presence of eleven currently known superantigens was determined in all the strains by multiplex PCR.

**Results:**

Totally, 124 beta-hemolytic streptococcal strains were isolated and they were classified as group A streptococcus (GAS) [15.3% (19/124)], group C streptococcus (GCS) [59.7% (74/124)] and group G streptococcus (GGS) [25.0% (31/124)]. Among 124 strains, only 35 strains were *emm *typeable and the remaining 89 strains were *emm *nontypeable. All GAS isolates were typeable, whereas most of the GCS and GGS strains were nontypeable. These nontypeable strains belong to *S. anginosus *[75.3% (67/89)] and *S. dysgalactiae *subsp. *equisimilis *[24.7% (22/89)]. The *emm *and *emmL *types identified in this study include *emm12.0 *(28.6%), *stG643.0 *(28.6%), *stC46.0 *(17.0%), *emm30.11 *(8.5%), *emm3.0 *(2.9%), *emm48.0 *(5.7%), *st3343.0 *(2.9%), *emm107.0 *(2.9%) and *stS104.2 *(2.9%). Various superantigen profiles were observed in typeable as well as nontypeable strains.

**Conclusions:**

Multiplex PCR analysis revealed the presence of superantigens in all the typeable strains irrespective of their *emm *types. However, the presence of superantigen genes in *emm *and *emmL *nontypeable strains has not been previously reported. In this study, presence of at least one or a combination of superantigen coding genes was identified in all the *emm *and *emmL *nontypeable strains. Thus, the superantigens may inevitably play an important role in the pathogenesis of these nontypeable strains in the absence of the primary virulence factor, M protein.

## Background

*Streptococcus pyogenes *(group A streptococcus) is a Gram-positive bacterial pathogen. Extracellular surface molecules such as the M protein, hyaluronic acid capsule and fibronectin-binding proteins allow group A streptococcus (GAS) to adhere to, colonise, and invade human skin and mucus membranes under different environmental conditions [1]. GAS is the causative agent of acute bacterial pharyngitis, scarlet fever and impetigo [2] and is responsible for a wide variety of life threatening diseases in humans. The chronic infections lead to acute rheumatic fever (ARF) and rheumatic heart disease (RHD). In recent years, the GAS infections and associated complications are emerging in developing countries like India. More than 60 GAS virulence factors were identified, which were responsible for adherence to epithelial cells, internalization, invasion, systemic toxicity, immune modulation and evasion, cellular or tissue disruption and bacterial transmission [3]. The major virulence factor reported in GAS is the M protein encoded by *emm *gene. The M protein confers resistance to phagocytosis by polymorphonuclear leukocytes [4] and binds to complement control factors and thereby prevents the activation of alternate complement pathway [1]. The GAS produces serum opacity factor (SOF) both in extracellular and membrane-bound form [5], which is involved in GAS-host cell adherence process [6].

In 1935, group C streptococcus (GCS) and group G streptococcus (GGS) were first identified as human pathogens [7]. The infections with GCS and GGS can also cause acute pharyngitis, necrotizing fasciitis, sepsis, soft tissue infections and streptococcal toxic shock-like syndrome [8]. *S. dysgalactiae *subsp. *equisimilis *and *S. anginosus *are the two important species of GCS/GGS groups. *Streptococcus dysgalactiae *subsp. *equisimilis *can cause the whole spectrum of infections caused by *S. pyogenes *[9,10]. A high rate of pharyngeal carriage of GCS and GGS in acute rheumatic fever (ARF) patients has also been reported [11].

GAS, GCS and GGS possess common virulence mechanisms including the expression of streptokinase, hyaluronidase, C5a peptidase and M protein [12]. Recently, the presence of GAS virulence genes encoding streptolysin S and glyceraldehyde-3-phosphate dehydrogenase has been reported in bovine *S. dysgalactiae *subsp. *dysgalactiae *(GCS) and human *S. dysgalactiae *subsp. *equisimilis *(GCS/GGS) [13]. Several reports indicated that GCS and GGS also possess *emm *or *emmL *genes [12,14]. The existence of a considerable level of *emm *gene sequence homology between GAS and GCS/GGS groups has been reported [15]. The *emmL *gene polymorphisms have also been identified in human GGS isolates [6]. Several *emm *and *emmL *types of streptococci have been reported in India [16].

GAS produces many pyrogenic exotoxins, called streptococcal pyrogenic exotoxins (SPEs). These toxins act as superantigens (SAgs), which are potent T cell mitogens. Superantigens simultaneously bind to MHC class II antigens and T cell receptor (TCR) molecules and stimulate T cells to increase the levels of the cytokines TNF-α and IL-1β and T cell mediators like IL-2 and IFN-γ [17]. These cytokines in high concentration cause fever and shock [18].

The present study describes the prevalence of *emm, emmL *and SAg genes in the pharyngeal GAS, GCS and GGS strains isolated from a hospital in Tamil Nadu State, India. An attempt was made to determine the relationship between the superantigen profiles and *emm *types of pharyngeal streptococci. The strains that possess *emm *or *emmL *genes are referred to as typeable strains and the strains lacking *emm *or *emmL *genes are referred to as nontypeable strains. The presence of superantigen genes has been previously reported in typeable GAS, GCS and GGS strains. The presence of two GAS superantigens, streptococcal mitogenic exotoxin Z (*smeZ*) and streptococcal superantigen (*ssa*) has been reported in GGS *emm *types, *st245 *and *stG480 *[19]. The presence of GAS superantigen, streptococcal pyrogenic exotoxin M (*speM*), is detected in GGS *emm *type, *stG10 *and GCS *emm *type, *stC1400 *[19-21]. However, the presence of superantigen genes in nontypeable strains has not been previously reported. Here, we report the occurrence of different superantigen genes in typeable as well as nontypeable strains of pathogenic streptococci.

## Methods

### Strain collection and identification

Bacterial strains were isolated over a period of one-year from January to December 2009 from Govt. District Headquarters Hospital, Virudhunagar, Tamil Nadu, India. A total of 1040 outpatients of Pediatrics Department between the age of 4 and 12 years with sore throat as their primary complaint were selected. Throat swabs were collected by swabbing the throat in the area of tonsils. The swabs were inoculated immediately on 5% sheep blood agar and the plates were incubated in 5 to 7% CO_2 _at 35°C for overnight. The isolates were Lancefield serogrouped using HiStrep latex test kit (Hi-Media Laboratories Pvt. Ltd., Mumbai, India) according to the manufacturer's instructions. Pyrrolidonylarylamidase (PYR) test was performed for the differentiation of *S. pyogenes *from *S. dysgalactiae *subsp. *equisimilis, S. equi *subsp. *zooepidemicus *and *S. anginosus*. Acid production from trehalose and sorbitol was used to differentiate *S. dysgalactiae *subsp. *equisimilis *from *S. equi *subsp. *zooepidemicus*. Voges-Proskauer test was done for the differentiation of *S. dysgalactiae *subsp. *equisimilis *and *S. equi *subsp. *zooepidemicus *from *S. anginosus *[10,22,23].

### Bacterial strains and culture conditions

Beta-hemolytic streptococcal isolates were grown in 5% sheep blood agar and stored in Todd-Hewitt broth with 20% glycerol at -80°C until used. *Escherichia coli *DH5α (F^- ^*end*A1 *gln*V44 *thi*-1 *rec*A1 *rel*A1 *gyr*A96 *deo*R *nup*G Φ80d*lacZ*ΔM15 Δ(*lacZYA-argF*) U169, *hsd*R17(rK^- ^mK^+^), λ-) was grown in LB broth or agar medium. LB medium supplemented with ampicillin (100 μg/ml) was used to select transformants and maintain plasmids.

### Antibiotic susceptibility test

Streptococcal isolates were screened for susceptibility to penicillin G (10 units), ampicillin (10 μg), amoxicillin (20 μg), erythromycin (15 μg), azithromycin (15 μg), tetracycline (30 μg), ceftazidime (10 μg) and ceftriaxone (30 μg) by Kirby-Bauer disc diffusion method on Mueller-Hinton agar supplemented with 5% sheep blood. *S. pyogenes *ATCC 12384 was used as a positive control. An overnight grown broth culture of test strain was adjusted to 0.5 MacFarland's turbidity standard, and then swabbed evenly on the culture plate. Antibiotic discs were placed on the plate and incubated at 37°C under an atmosphere containing 5-7% CO_2 _for 18 h. Results were interpreted according to the Clinical Laboratory Standard Institute (CLSI) guidelines [24].

### Serum opacity factor (SOF) typing

Serum opacity factor typing of the streptococcal isolates was done using horse serum according to the method of WHO [25].

### DNA extraction and PCR amplification of *emm *and *emmL *genes

Genomic DNA was extracted from all the beta-hemolytic streptococci by using spin column kit following manufacturer's instructions (Hi-Media Laboratories Pvt. Ltd., Mumbai, India). The *emm *gene was amplified with the "all M" primers [26] and *emm*2F-*emm*2R primers [27] (Table 1). The *emmL *gene was amplified with G1F and G1R primers [6]. All the 124 beta-hemolytic streptococci were subjected to PCR using these three primer pairs. PCR reactions were carried out in an Eppendorf mastercycler (Eppendorf AG, Hamburg, Germany) with 35 cycles, each cycle consisting of initial denaturation at 94°C for 5 min, denaturation at 94°C for 30 s, annealing at 52°C for 30 s, elongation at 72°C for 2 min and a final elongation at 72°C for 5 min. PCR products were resolved on 1% agarose gel, stained with ethidium bromide and analyzed in gel documentation unit (Bio-Rad, USA).

**Table 1:** List of primers used in this study

Primer	Sequence (5'-3')	Reference
***emm*/*emmL *typing** **primers**		
*emm *1"all M" - F	GGGGGGGGATCCATAAGGAGCATAAAAATGGCT	[26]
*emm *1"all M"- R	GGGGGGGAATTCAGCTTAGTTTTCTTCTTTGCG	
*emm *2 - F	TATTSGCTTAGAAAATTAA	[27]
*emm *2 - R	GCAAGTTCTTCAGCTTGTTT	
*emmL *- G1F	AAAAATAAGGAGAAAAAATGG	[5]
*emmL *- G1R	TTTTTAGTTTTCTTCTTTGCG	
**Superantigen primers**		
*speC *- F	GGTAAATTTTTCAACGACACACACATTAAA	
*speC *- R	TGTTGAGATTCTCCCGAAATAAATAGAT	
*speG *- F	GCTATGGAAGTCAATTAGCTTATGCAGAT	
*speG *- R	TTATGCGAACAGCCTCAGAGG	
*speJ *- F	CAATTAAATTACGCATACGAAATCATACCAGTA	
*speJ *- R	ACGAGTAAATATGTACGGAAGACCAAAAATA	
*speK *- F	TATCGCTTGCTCTATACACTACTGAGAGT	
*speK *- R	CCAAACTGTAGTATTTTCATCCGTATTAAA	[21]
*speL *- F	GGACGCAAGTTATTATGGATGCTCA	
*speL *- R	TTAAATAAGTCAGCACCTTCCTCTTTCTC	
*speA1-3,5*-F	GGTATTTGCTCAACAAGACCCCGAT	
*speA1-3,5*-R	TGTGTTTGAGTCAAGCGTTTCATTATCT	
*speA1-4*-F	CAAGAAGTATTTGCTCAACAAGACCCCA	
*speA1-4*-R	TTAGATGGTCCATTAGTATATAGTTGCTTGTTATC	
*speH *-F	TCTATCTGCACAAGAGGTTTGTGAATGTCCA	
*speH *-R	GCATGCTATTAAAGTCTCCATTGCCAAAA	
*speI *-F	AAGGAAAAATAAATGAAGGTCCGCCAT	
*speI *-R	TCGCTTAAAGTAATACCTCCATATGAATTCTTT	
*speM *-F	GCTTTAAGGAGGAGGAGGTTGATATTTATGCTCTA	
*speM *-R	CAAAGTGACTTACTTTACTCATATCAATCGTTTC	
*smeZ-*F	CAATAATTTCTCGTCCTGTGTTTGGAT	
*smeZ-*R	GATAAGGCGTCATTCCACCATAG	
*ssa *-F	AATTATTATCGATTAGTGTTTTTGCAAGTA	
*ssa *-R	AGCCTGTCTCGTACGGAGAATTATTGAACTC	

### *emm *and *emmL *typing

All the amplicons were individually cloned in a PCR cloning vector, pTZ57RT (MBI-Fermentas, St. Leon-Rot, Germany) and sequenced employing universal M13 and M13R primers at Macrogen, Seoul, South Korea. The *emm *and *emmL *gene sequences were aligned online with sequences available at the CDC database http://www.cdc.gov/ncidod/biotech/strep/strepblasts.html to identify the *emm *types.

### Dot-blot analysis

The absence of *emm *or *emmL *genes in the genomes of nontypeable strains were confirmed by dot-blot hybridization using complete *emm *and *emmL *genes as probes. Genomic DNA (100 ng) of all the nontypeable strains were spotted on nylon membranes (Biodyne^® ^A, Pall, USA) and immobilized by UV-cross linking. The complete *emm *and *emmL *genes were labeled with biotin using NEBlot phototype kit (New England BioLabs, MA, USA) and used as the probe. Hybridization and washing steps were performed as per the manufacturer's instructions. Genomic DNA samples of typeable GAS, GCS and GGS isolates were used as positive controls. Further, the membrane was processed with NEB Phototope^®^-Star detection kit to detect the presence or absence of *emm *and *emmL *homologs. The luminescence was visualized using ChemiDoc imaging system (UVP, UK).

### Multiplex PCR for superantigens

Multiplex PCR was performed using Qiagen multiplex PCR kit (Qiagen, Hilden, Germany) to check the presence of streptococcal pyrogenic exotoxin C (*speC*), *speG, speJ, speK, speL, speH, speI, speM, speA*, streptococcal mitogenic exotoxin Z (*smeZ*) and streptococcal superantigen (*ssa*) in all the streptococcal isolates with the reported primers [21] (Table 1). PCR reactions were performed in an Eppendorf mastercycler with 35 cycles with each consisting of an initial denaturation at 95°C for 15 min, denaturation at 94°C for 30 s, annealing at 57°C for 90 s, elongation at 72°C for 90 s and final elongation at 72°C for 10 min. The reaction mixture consisted of 25 μl of 2X Qiagen multiplex PCR master mix, 2 mM of primer mix (adjusted to 5 μl), 200 ng of template DNA (adjusted to 5 μl) and the final volume was adjusted to 50 μl using sterile deionized water. PCR products were resolved on a 2% (w/v) agarose gel and analyzed as described above.

### Nucleotide submissions

The identified *emm *and *emmL *gene sequences were submitted to GenBank database under the accession numbers HM125069-HM125081, HM449040-HM449043.

## Results

### Characteristics of streptococcal isolates

In the present study, 124 beta-hemolytic streptococcal strains were isolated and they were classified as group A streptococcus (GAS) [15.3% (19/124)], group C streptococcus (GCS) [59.7% (74/124)] and group G streptococcus (GGS) [25.0% (31/124)]. Within GCS strains, 22 isolates were identified as *S. dysgalactiae *subsp. *equisimilis *and 52 as *S. anginosus*. In GGS, 16 isolates were identified as *S. dysgalactiae *subsp. *equisimilis *and 15 were identified as *S. anginosus *(Table 2). Antibiotic susceptibility pattern of all 124 streptococcal strains revealed that all of them were sensitive to penicillin G and all other tested antibiotics.

**Table 2:** *emm *and *emmL *typeable and nontypeable pharyngeal streptococci isolated in this study

*emm *or *emmL *type	No. of isolates of:					
		
	*S. pyogenes*	*S. dysgalactiae* subsp. *equisimilis*		*S. anginosus*		
		
	GAS	GCS	GGS	GCS	GGS	
*emm12.0*	10					10 (8.1)
*stG643.0*			10			10 (8.1)
*stC46.0*		6				6 (4.8)
*emm30.11*	3					3 (2.4)
*emm3.0*	1					1 (0.8)
*emm48.0*	2					2 (1.6)
*st3343.0*	1					1 (0.8)
*emm107.0*	1					1 (0.8)
*stS104.2*	1					1 (0.8)
Nontypeable	0	16	6	52	15	89 (71.8)
Subtotal	19	22	16	52	15	
Total for	19		38		67	124
species						

GAS, Group A streptococci; GCS, Group C streptococci; GGS, Group G streptococci.

### *emm *and *emmL *typing

Among the 124 strains analyzed, only 35 of the strains were found to be positive for either *emm *or *emmL *typing identified by the PCR amplification of *emm *or *emmL *genes (0.9 to 1.5 kb) as reported earlier [28]. Of these 35 strains, 19 (54.3%) were positive for *emm *gene, while 16 (45.7%) were positive for *emmL *gene. All the 19 GAS strains were *emm *positive and hence these strains were considered *emm *typeable. Of the 16 *emmL *positive strains, 10 strains were of GGS and the other 6 were GCS and these strains could be considered as *emmL *typeable. However, the majority of the isolates [89/124 (71.8%)] were negative for both *emm *and *emmL *genes. Further, to examine the presence of *emm *or *emmL *homologs in their genomes, the dot-blot hybridization was performed with *emm *and *emmL *gene specific probes and genomic DNA. However, no detectable signal was obtained suggesting that these strains were truly *emm *nontypeable. Of these 89 nontypeable strains, 22 (24.7%) were identified as *S. dysgalactiae *subsp. *equisimilis *and 67 (75.3%) were *S. anginosus*. Within the 22 strains of *S. dysgalactiae *subsp. *equisimilis*, 16 strains were of GCS and 6 strains were of GGS. Among the 67 strains of *S. anginosus*, 52 were GCS and 15 were of GGS (Table 2).

The *emm *or *emmL *typeable strains were subjected to RFLP analysis to ascertain the *emm *type. RFLP analysis of these 35 strains revealed 9 distinct patterns (data not shown). All the amplicons regardless of their RFLP pattern were cloned and sequenced. Sequence analysis revealed that RFLP pattern and sequences were similar, thus representing 9 *emm *types among the 35 typeable strains (Table 3).

**Table 3:** Superantigen profiles of *emm *and *emmL *typeable and nontypeable strains of pharyngeal streptococci.

S.No	Superantigen Profiles	No. of typeable strains	No. of nontypeable strains
1.	*speC*	0	1
2.	*speC+ smeZ*	0	6
3.	*speC+ speG*	3 (*emm 30.11*)	0
4.	*speC+ speA1-3,5*	1 (*st3343.0*)	0
5.	*speC+ speG+ speA1-4*	2 (*emm 48.0*)	3
6.	*speC+ speG+ speA1-3,5*	1 (*emm 107.0*)	1
7.	*speC+ speG+ speL*	0	3
8.	*speC+ speG+ smeZ*	0	15
9.	*speC+ speA1-4+ speA1-3,5*	0	1
10.	*speC+ speA1-4+ smeZ*	0	4
11.	*speC+ speG+ speA1-4+ speA1-3,5*	1 (*emm3.0*)	3
12.	*speC+ speG+ speA1-4+ smeZ*	10 (*stG643.0*)	27
13.	*speC+ speG+ speL + smeZ*	0	6
14.	*speC+ speG+ speL+ speI*	0	1
15.	*speC+ speG+ speL+ speA1-4+ smeZ*	10 (*emm12.0*)	7
16.	*speC+ speG+ speA1-4+ speA1-3,5+ smeZ*	0	1
17.	*speC+ speG+ speA1-4 + speH+ smeZ*	0	1
18.	*speC+ speG+ speA1-4+ speA1-3,5+ speI*	0	1
19.	*speC+ speG+ speH+ smeZ+ speI*	0	1
20.	*speC+ speG+ speA1-4+ smeZ+ speI*	0	1
21.	*speC+ speA1-4+ speH + smeZ+ speI*	0	2
22.	*speC+ speG+ speA1-4+ speA1-3,5+ speH+ smeZ*	0	1
23.	*speC+ speG+ speA1-4+ speA1-3,5+ speH+ speI*	0	1
24.	*speC+ speG+ speL+ speA1-4+ smeZ+ speI*	1 (*stS104.2*)	0
25.	*speC+ speG+ speA1-4+ speH+ smeZ+ speI*	0	1
26.	*speC+ speG+ speA1-4+ speA1-3,5+ speH+smeZ+ speI*	6 (*stC46.0*)	1

### Serum opacity factor (SOF)

Among the 35 *emm *and *emmL *typeable strains, 5 of the GAS strains that belong to *emm48.0, emm107.0 *and *stS104.2 *were SOF-positive and the other 30 strains were SOF-negative. Significantly, all the nontypeable GCS and GGS strains were SOF-negative.

### Superantigen profiling

The superantigen profiles (combinations of superantigen genes) of the 124 isolates are summarized in Table 3. The superantigen, *speC*, was detected in all the strains, whereas *speJ, speK, speM *and *ssa *were detected in none of the isolates. Among the 19 GAS typeable strains, 10 *emm12 *type strains were positive for *speC, speG, speL, speA1*-*4 *and *smeZ*. Ten GGS typeable strains (*stG643.0*) were positive for *speC, speG, speA1*-*4 *and *smeZ*. Six GCS typeable strains (*stC46.0*) were positive for six superantigens (*speC, speG, speA, smeZ, speH *and *speI*). Interestingly, all nontypeable strains were also found to have at least one superantigen gene (Table 3). Among nontypeable strains, 27 were positive for *speC, speG, speA1*-*4 *and *smeZ *and 15 strains were positive for *speC, speG *and *smeZ*. Maximum of six superantigen genes (*speC, speG, speA, smeZ, speH *and *speI*) were detected in a single GCS nontypeable strain (*S. anginosus*). These results revealed that all the typeable and nontypeable pathogenic beta-hemolytic streptococcal isolates possess at least one superantigen gene.

## Discussion

In the present study, a total of 124 strains were isolated from acute pharyngitis patients. Interestingly, 71.8% of them were *emm *and *emmL *nontypeable. The *emm12 *was the predominant *emm *type (28.6%) among the typeable GAS strains. The prevalence of *emm12 *was previously reported among the pharyngeal isolates of GAS from Chennai, South India [29]. The presence of *emm1, emm3, emm28, emm12, emm4 *and *emm11 *has been reported among the North Indian GAS isolates [30].

In this study, *stG643.0 *was the predominant *emm *type (28.6%) among GGS strains. In contrast, two other GGS *emm *types, *stG485 *and *stGLP1*, were previously reported to be prevalent among 49 GGS isolates from Chennai, India [31]. Other *emm *types previously reported from India [8] which also represented in this study were *emm3 *(2.9%) and *stC46 *(17.0%). In addition, the newly identified *emm *types include *emm48.0 *(5.7%), *st3343.0 *(2.9%), *emm107.0 *(2.9%), *stS104.2 *(2.9%), and *emm30.11 *(8.5%). According to CDC, these *emm *types have already been reported in United States, Mexico, Malaysia and New Zealand, but not from India. Therefore, further studies on the epidemiology of these *emm *types in India are needed.

The emergence of drug resistance among streptococci to macrolides (erythromycin and clarithromycin) and tetracycline are widely reported in recent years [32-34]. However, in the present study, all the streptococcal strains were susceptible to all the tested antibiotics including β-lactams, macrolides and tetracycline. Therefore, drug resistance is not a major threat among the streptococci isolated in this study population. However, untreated chronic infections may lead to severe complications such as acute rheumatic fever (ARF) and rheumatic heart disease (RHD).

SOF acts as an adhesin involved in the adherence of GAS to host cells [35]. It has also been reported that the interaction between SOF and the host extracellular matrix protein, fibulin-1, may be involved in the adhesion of GAS to extracellular matrices of the host [36]. Courtney et al. [37] reported that the SOF was expressed at a frequency of 50% among the clinical isolates of GAS. In contrast, only 14.3% of typeable GAS isolates were positive for SOF and all GCS and GGS strains were SOF-negative. Similarly, McDonald et al. [15] reported that most of the GCS and GGS strains of *S. dysgalactiae *subsp. *equisimilis *were SOF-negative. Therefore, SOF may not be the only factor responsible for the adhesion of GAS to the host and some other mechanisms might exist [37]. Some of the GAS adhesins reported other than SOF include lipoteichoic acid, hyaluronic acid capsule, vitronectin-binding protein and collagen-binding protein [2].

We have observed significant differences in the distribution of superantigen genes between typeable and nontypeable strains. The co-occurance of number of superantigen genes was relatively more in typeable than in nontypeable strains. Among 11 superantigens, a maximum of 6 superantigens (*speC, speG, speA, speH, speI *and *smeZ*) was detected in 17.1% of typeable strains, whereas this combination was detected in only 1.1% nontypeable strains. Totally, 26 superantigen profiles have been identified among the typeable and nontypeable strains. Of these, 23 profiles were detected in nontypeable strains and 9 profiles in typeable strains. Only six superantigen profiles were identified in both typeable and nontypeable strains. Other 20 profiles were confined to either typeable or nontypeable strains. As reported previously [38], the most prevalent chromosomally encoded superantingens, *speG *and *smeZ*, were detected in 97.1% and 77.1% of the typeable strains, respectively. In addition, these two genes were detected in 84.3% and 83.1% of nontypeable isolates, respectively. Similarly, Proft et al. [39] have reported the presence of these two superantigens in all the strains of GAS.

A high occurrence of GCS (59.7%) and GGS (25.0%) among pharyngeal streptococcal isolates was observed, which is in agreement with previous reports from Spain and Australia [10,15]. It is known that more virulent forms of GCS and GGS could have emerged through the acquisition of superantigen genes from GAS [19]. In this study, many superantigen profiles have been identified among GCS and GGS strains and these strains could be considered as emerging pathogens.

Overall, 67 *S. anginosus *(52 GCS and 15 GGS) strains and 22 *S. dysgalactiae *subsp. *equisimilis *(16 GCS and 6 GGS) strains were nontypeable. Although the presence of superantigens has been reported in typeable GCS and GGS isolates, their presence in nontypeable strains has not been reported so far. However, in our study, compared to the strains of typeable *S. pyogenes *(GAS) and typeable as well as nontypeable *S. dysgalactiae *subsp. *equisimilis*, more numbers of superantigen profiles have been observed in nontypeable strains of *S. anginosus *(GCS/GGS). In a very recent study [13], the presence of GAS superantigens (*speL, speC, speK *and *speM*) has been reported in typeable *S. dysgalactiae *subsp. *dysgalactiae *(GCS) strains associated with bovine mastitis, but not in the human isolates of *S. dysgalactiae *subsp. *equisimilis *(GCS/GGS). We have identified the presence of superantigens in nontypeable strains of *S. dysgalactiae *subsp. *equisimilis *(GCS/GGS) associated with acute pharyngitis in humans. Therefore, our findings suggest that, like *S. pyogenes, S. dysgalactiae *subsp. *equisimilis *and *S. anginosus *may emerge as potential human pathogens. Some other factors might be involved in the pathogenesis of nontypeable strains in the absence of M protein. The presence of superantigens in nontypeable strains has not been reported so far. The role of superantigens in the pathogenesis of typeable GCS and GGS strains is well documented [2,19,20]. However, the role of superantigens in the pathogenic mechanisms of nontypeable strains is yet to be studied.

## Conclusions

A high occurrence of GCS and GGS strains of *S. anginosus *and *S. dysgalactiae *subsp. *equisimilis *was observed among the acute pharyngitis patients and most of them were nontypeable. In addition, the presence of superantigens in nontypeable GCS and GGS strains was observed for the first time. Therefore, these strains may emerge as potential human pathogens. Though antibiotic resistance was not observed among these strains, earlier diagnosis and proper antibiotic treatment are required to cure acute pharyngitis and prevent from subsequent complications such as acute rheumatic fever (ARF) and rheumatic heart disease (RHD).

## Competing interests

The authors declare that they have no competing interests.

## Authors' contributions

PG designed and coordinated the entire work. TD collected clinical samples and isolated pharyngeal streptococcal strains. TD and TR carried out *emm *typing. TD and JR carried out superantigen profiling. TD and JR wrote the manuscript and PG corrected the manuscript. All authors have read and approved the final version of the manuscript.
